# Prognostic Value of High CXCR4 Expression in Renal Cell Carcinoma: A System Review and Meta-Analysis

**DOI:** 10.1155/2015/568980

**Published:** 2015-10-07

**Authors:** Yuefeng Du, Qingzhi Long, Bing Guan, Lijun Mu

**Affiliations:** Department of Urology, First Affiliated Hospital of Medical School, Xi'an Jiaotong University, Xi'an, Shaanxi, China

## Abstract

*Background*. Recent studies have shown that CXC chemokine receptor 4 (CXCR4) is involved in the progression and metastasis of renal cell carcinoma (RCC). However, the prognostic value of CXCR4 expression in RCC remains controversial. The aim of our meta-analysis is to evaluate the prognostic value of high CXCR4 expression in RCC. *Methods*. Relevant studies focused on the relationship between high CXCR4 expression and the outcome of RCC were searched in PubMed and EMBASE/Cochrane Library database. Hazard ratios (HRs) of overall survival (OS) and progression-free survival (PFS) were our evaluation index. The individual and pooled HRs with 95% confidence intervals (CIs) were analyzed. *Results*. A total of 1068 patients from 7 studies were included in our meta-analysis. The results suggested that high CXCR4 expression predicted a poor OS (random effect model (REM) HR = 2.77, 95% CI = 1.80−4.27) and PFS (REM HR = 4.83, 95% CI = 2.30−10.15) for RCC patients. *Conclusion*. The results of meta-analysis indicated that high CXCR4 expression was correlated with worse OS and PFS for patients with RCC. However, some larger samples and well-matched studies should be designed to estimate the potential prognosis of RCC patients.

## 1. Introduction

Renal cell carcinoma (RCC), which is the fifth most common cancer worldwide, accounts for 2-3% of all malignant diseases in adults [1, 2]. Although an increasing number of patients with small and early stage RCC are diagnosed, there are approximately 30% of patients developing to metastatic disease and usually leading to incurable disease [3, 4]. The patients with advanced stage RCC had a low two-year survival of 8% because of chemo- and radioresistance and the cytokines toxicity profile [5–7]. Once metastasis occurred, most of the patients would relapse and finally die of the disease regardless of tyrosine-kinases and mTOR-inhibitors had been used in clinic [8, 9]. Nevertheless, it is not active for all patients and these drugs are expensive. Thus, it is very necessary to identify prognostic and predictive markers and to develop more effective systemic therapies for different patients.

CXC chemokine receptor 4 (CXCR4) is one of 19 acknowledged chemokine receptors and belongs to the family of G-protein coupled chemokine receptor [10]. Currently, CXCR4 had been proved to be overexpressed in over 20 human malignancies, such as thyroid cancer, breast cancer, pancreatic cancer, prostate cancer, and kidney cancer [11, 12]. The results of some studies suggested that overexpression of CXCR4 which was detected in resected primary tumor tissues was associated with distal metastasis and poor prognosis [13, 14]. Wang et al. and Zhao et al. had demonstrated that high expression of CXCR4 was strongly related to poor survival of patients with metastatic RCC [15, 16]. Some other studies also recommended CXCR4 plus other chemokine receptors as the new biomarkers for prognosis of patients with RCC [17, 18]. However, the prognosis value of CXCR4 is controversial because of insufficient samples and limited studies. So, we performed this meta-analysis to systematically and comprehensively evaluate the prognosis value of CXCR4 in outcome of patients with RCC.

## 2. Materials and Methods

### 2.1. Study Selection

Two independent authors comprehensively searched the PubMed and EMBASE/Cochrane Library for relevant articles published up to June 1, 2015. The key terms included renal cell carcinoma (“renal cancer” OR “renal cell carcinoma” OR “renal carcinoma” OR “renal tumor”), CXCR4 (“CXCR4” OR “chemokine receptor 4”), prognosis, and survival. The language of articles was limited to English. In addition, we also checked reference lists of identified studies for the other potential eligible trials. This progress was stopped when there were not additional articles.

### 2.2. Inclusion and Exclusion Criteria

The eligible studies included in this meta-analysis must met the following criteria to reduce the heterogeneity of articles: (1) patients with distinctive renal cell carcinoma diagnosis by pathology without the limitation of age and gender; (2) using immunohistochemistry method to detect the expression of CXCR4; (3) articles focused on the association of high CXCR4 expression and poor prognosis of patients with RCC; and (4) articles having the hazard ratios (HRs) of overall survival (OS) or progression-free survival (PFS) about CXCR4 expression and survival. The case reports, letters, and expert opinions were excluded. The exclusion criteria of studies also included (1) articles about cell lines or animals; (2) no definition of expression of CXCR4; (3) articles' lack of original data and control groups; (4) no relevant outcome data of OS or PFS; and (5) repetitive articles.

### 2.3. Data Abstraction and Quality Assessment

Relevant characteristics and outcome data were collected by two independent reviewers. The main characteristics of articles were listed as follows: (1) first author's name; (2) publication year; (3) country; (4) study period; and (5) median follow-up. The relevant clinical data of studies included (1) patients' number; (2) gender (male/female); (3) age (years); (4) pathological pattern; (5) Fuhrman grade; (6) histologic origin; (7) antibody source; (8) dilution; (9) evaluation method of CXCR4 expression level; and (10) low versus high CXCR4. The HR with 95% confidence interval (CI) was the outcome data. If an article provided the results of univariate and multivariate analyses, we chose the latter. Any disagreement was resolved by discussion. The quality of articles included in this meta-analysis was assessed by Newcastle-Ottawa Scale (NOS).

### 2.4. Data Analysis

A pooled analysis of HRs and 95% CIs was used to evaluate the effect of CXCR4 expression on the survival of renal cell carcinoma in this meta-analysis. Subgroup analyses were conducted according to the ethnicity, the proportion of Fuhrman grade III-IV patients, and duration of follow-up. If the number of studies is not insufficient in subgroup analysis, then it will be listed simply. We further conducted sensitivity analyses to detect the possible reasons for heterogeneity and to evaluate the effect of each study on the overall pooled estimate. Chi square (*χ* ^2^) and *I* ^2^ statistics values were used for assessment of heterogeneity. There was a significant difference in survival between high and low of CXCR4 expression when *P* < 0.05, except for special instructions. Fixed effect was used for meta-analysis if the value of *I* ^2^ > 50%. Otherwise, we would choose the random effect. The potential publication bias was evaluated by visually symmetry of Begg funnel plots. Moreover, Begg and Egger tests were used for the quantification of publication bias. STATA 12.0 software was used for all statistical analyses if there are no special instructions.

## 3. Results

### 3.1. Study Characteristics

**Figure 1:**
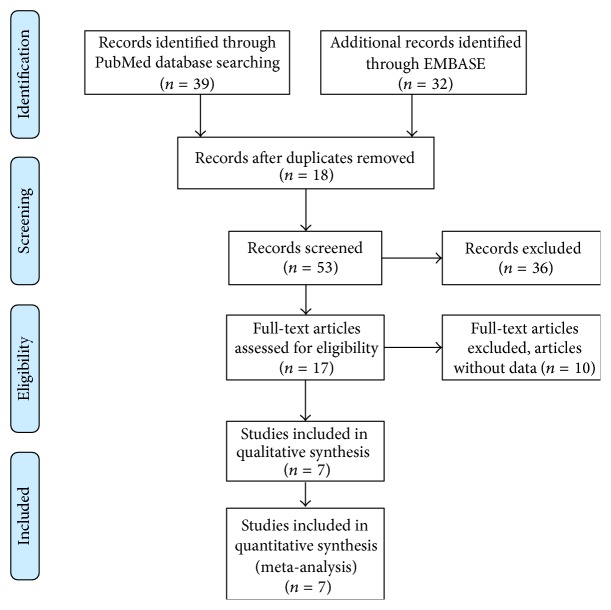
Flow diagram for articles included in this meta-analysis.

There were 7 relevant articles finally included in our meta-analysis [2, 11, 17–21]. The details of selection process were shown in Figure 1. These studies were conducted in 3 countries published between 2010 and 2014 (China, France, and Italy). The total number of patients was 1068, and the sample sizes ranged from 62 to 240 patients. The median follow-up period ranged from 28.8 to 79.2 months. The main characteristics and NOS scores of articles were listed in Table 1. The baseline information and clinical data of studies included in this meta-analysis were listed in Table 2.

### 3.2. High CXCR4 Expression and Prognosis of RCC

**Figure 2:**
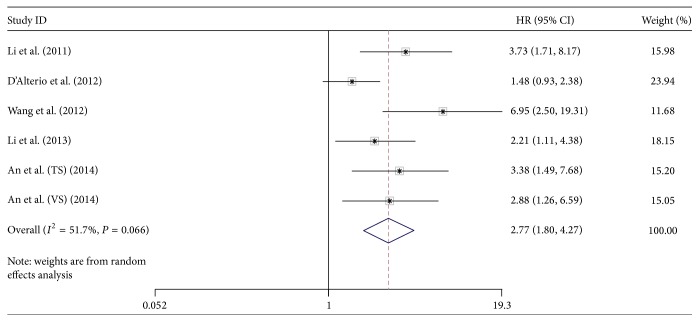
The forest plot of HRs for OS with 5 studies included in this meta-analysis.

**Figure 3:**
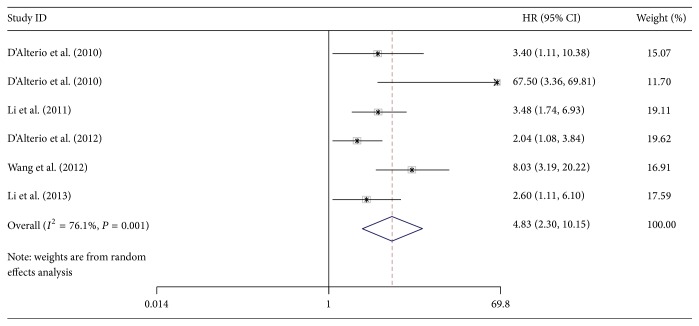
Forest plot of HRs for PFS with 6 studies included in this meta-analysis.

**Figure 4:**
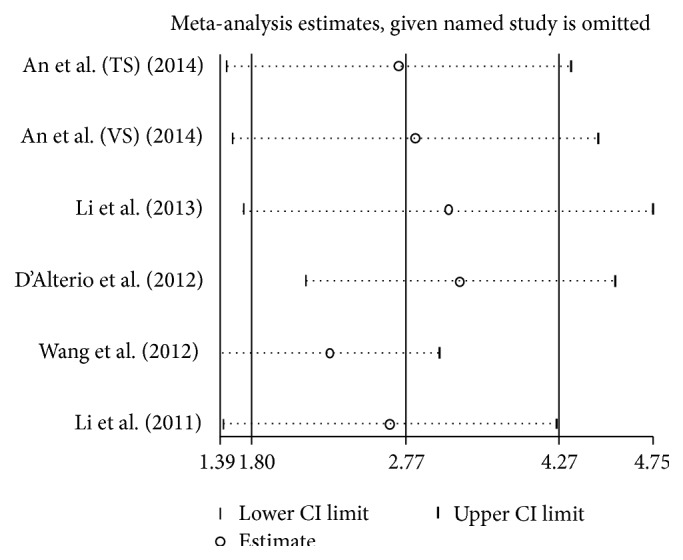
The plot of result of sensitivity analysis for OS.

**Figure 5:**
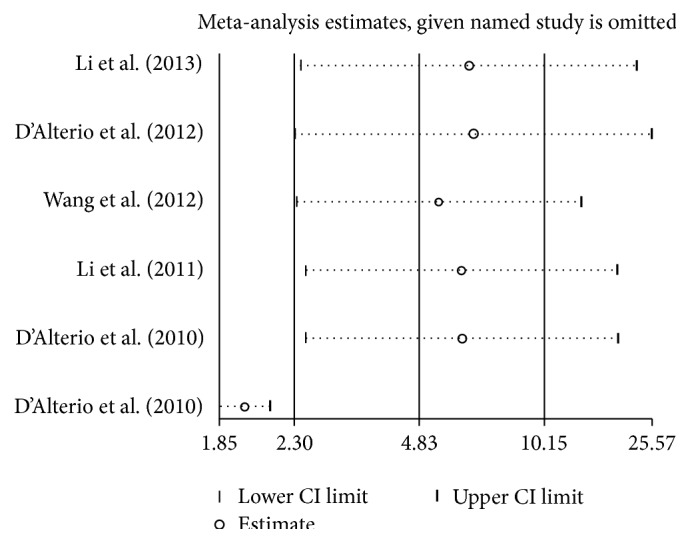
The plot of result of sensitivity analysis for PFS.

There were 5 articles involved with 605 patients providing the HR of OS. The result of meta-analysis showed that high CXCR4 expression predicts a poor OS (random effect model (REM) HR = 2.77, 95% CI = 1.80–4.27) with obvious heterogeneity (*I* ^2^ = 51.7%, *P* = 0.066) (Figure 2). There were 6 articles involved with 843 patients investigating the HR of PFS. The results of meta-analysis showed that high CXCR4 expression also predicts a poor PFS (REM HR = 4.83, 95% CI = 2.30–10.15) with significant heterogeneity (*I* ^2^ = 76.1%, *P* = 0.001) (Figure 3). The results of sensitivity analyses showed that when the studies eliminated in turn would not change the results of pooled analyses of OS (Figure 4) and PFS (Figure 5).

### 3.3. Subgroup Analysis

The association between high expression and poor OS was similar in Asian patients and non-Asian patients when grouped by ethnicity and median follow-up period. When grouped by the proportion of Fuhrman grade III-IV patients with RCC, it seemed that there was no association between high CXCR4 expression and poor OS (HR = 1.48, 95% CI = 0.93–2.37). However, only one article was involved. The results of subgroup analyses were shown in Table 3.

### 3.4. Publication Bias

The interpretability of publication bias assessed by Begg and Egger tests was limited when only 7 studies were included in this meta-analysis.

## 4. Discussion

Meta-analytical technique is qualitative and quantitative tool to evaluate those subjects which are still controversial. The results of meta-analysis always were regarded as the highest level of evidence. Nowadays, people do not fully understand which factors affect the prognosis of RCC patients. Many studies had been identifying the suitable molecular biological prognostic markers for RCC. Recently, a series of studies focused on the relationship of CXCR4 expression levels and the prognosis of patients with RCC. However, these studies did not achieve consensus. This is the first meta-analysis performed to elucidate the prognosis value of high CXCR4 expression in OS and PFS of RCC patients. The results of our meta-analysis suggested that high CXCR4 expression predicted poor OS and PFS. The CXCR4 may serve as a useful prognosis marker and a therapeutic target for the RCC.

Chemokines are peptide mediators involved in normal development, immune and hematopoietic regulation, inflammation, and wound healing [10]. CXCR4 is a kind of G-protein coupled chemokine receptor, which is always lowly or absently expressed in many normal tissues, including breast and ovary [11]. Previous studies had indicated that CXCR4 was involved in the vascularization and metastasis of cancer [15, 22]. CXCR4 had been proved to be upregulated in many cancers, including RCC and ovarian cancer [23]. The immunochemical method showed that CXCR4 expression mainly was in cytoplasm or membrane of tumor cells of clear cell RCC (ccRCC) [24]. The precise of molecular regulation mechanism of CXCR4 in RCC remains to be determined. The protein pVHL coded by VHL gene may have played a key role in the mechanism of metastasis in ccRCC. The protein pVHL has the capacity to degrade hypoxia-inducible factor (HIF) under normoxic conditions [25]. This process can be suppressed under a hypoxic condition, resulting in a HIF-dependent CXCR4 activation. Gahan et al. considered that CXCR4 may be crucial in controlling tumor cell adhesion via its interactions with integrin receptors [26]. Several researches also had demonstrated the importance of signaling of CXCR4 in cell-cycle regulation and apoptosis of renal cancer cells [20, 27]. The chemokine CXCL12 (SDF-1), which is now known as an exclusive ligand of CXCR4, regulates leukocyte precursor homing to bone marrow and other sites [10]. Many studies had demonstrated that CXCR4/CXCL12 axis plays an important role in regulating metastasis of CXCR4 positive tumor cells to the organs expressing CXCL12 [11, 27]. High CXCR4 expression may affect the chemotherapy drug reaction in metastatic renal cancer. Research showed that high CXCR4 expression was correlated with a sunitinib poor response for patients with metastatic renal cancer [19]. Guo et al. also considered that patients with negative or low CXCR4 expression were more likely to obtain longer PFS [28].

A series of meta-analyses had investigated the prognosis value of CXCR4 for patients with other system tumors. Han et al. considered that CXCR4 could help predict prognosis of gastric cancer patients [29]. The results of meta-analyses from Liu et al. indicated that high CXCR4 expression was associated with poor prognosis in ovarian cancer [30]. A growing body of evidence demonstrated that CXCR4 was not the only predictor of RCC. The meta-analysis of Wu et al. considered that systemic inflammatory response predicted a poor survival in patients with RCC [31]. D'Alterio et al. suggested that CXCR4 combined with CXCR7 was valuable prognostic factor in RCC patients [18].

However, the results of this meta-analysis need to be interpreted cautiously due to some limitations. First, only 7 relevant articles were only involved with 1068 patients and designed in Asia and Europe. Moreover, studies in other languages were excluded except for English, so language bias may exist in our meta-analysis. Second, there was a significant heterogeneity between these studies. The clinical characteristics of patients in each study such as age, gender, and performance status would lead to bias obviously. Third, the variable histologic type and immunohistochemistry method might affect the accuracy of this meta-analysis. Further researches should be conducted to investigate whether these factors would affect the results of meta-analysis.

## Figures and Tables

**Table 1:** Main characteristics and NOS score of each study included in meta-analysis.

First author	Year	Country	Study period	Median follow-up (year)	NOS score
An [2]	2014	China	1999–2006	TS: 5.2 (0.6–9.7) VS: 5.7 (0.7–9.8)	9
Li [20]	2013	France	1999–2005	6.6 (1.0–15.3)	8
D'Alterio [19]	2012	Italy	2005–2009	2.4	8
Wang [21]	2012	China	2002-2003	NA	7
Li [11]	2011	China	2001–2005	4.3 (0.2–8.3)	8
D'Alterio [17, 18]	2010	Italy	1999–2007	5.8	7
D'Alterio [17, 18]	2010	Italy	NA	5.3	8

TS: training set, VS: validation set, and NA: not available.

**Table 2:** Clinical data of studies included in meta-analysis.

First author/ publication year		Samples	Gender (M/F)	Age (years)	Pathological pattern	Fuhrman stage	Immunohistochemistry			Evaluation of expression level (CXCR4)	Low versus high (CXCR4)
							Histologic origin	Antibody source	Dilution		
An, 2014 [2]	TS VS	125 100	84/41 65/35	57.6 ± 12.7 60.5 ± 11.5	ccRCC ccRCC	I-II/III-IV; 75/50 I-II/III-IV; 62/38	Tumor sections	Mouse; R&D Systems, USA	1 : 400	Five-staged score (0, 1, 2, 3, and 4)	High (scores 0, 1, and 2) versus low (scores 3 and 4)

Li, 2013 [20]		104	69/35	64.5 (34–86)	ccRCC	I-II/III-IV; 63/41	Tissue sections	Rabbit; Abcam, UK	NA	Range of positive cells: high; moderate 25–85%; low <25%; and absence = 0%	≥85% versus <85%

D'Alterio, 2012 [19]		62	45/17	55 (31–82)	mRCC	Fuhrman grading: I-II/III-IV; 12/50	Histologic sections	Mouse; R&D Systems, USA	1 : 1000	The rate of stained (positive) tumor cells: 0–5% low, >5–20% intermediate, and >20% high	Negative/low versus Mediate versus high

Wang, 2012 [21]		97	60/37	≤60/>60; 63/34	ccRCC and others	Fuhrman grading: I-II/III-IV; 67/30	Tissue sections	Mouse; R&D Systems, USA	NA	The 25th percentile value of the average percentage of positive tumor cells	High (≥30%) versus low (<30%)

Li, 2011 [11]		117	78/39	≥60/<60; 54/63	ccRCC and LARCC	Fuhrman grading: I–III/IV; 96/21	Tissue sections	Mouse; R&D Systems, USA	1 : 100	Conventional four-tiered semiquantitative scoring system: scores 0–3 for negative, weak, moderate, and strong staining	Positive (+) versus negative (−)

D'Alterio, 2010 [17, 18]		223	121/102	≥70/<70; 99/124	RCC (chromophobe, conventional, papillary, bellini and other)	Fuhrman grading: I–III/IV; 168/53	Histologic sections	Mouse; R&D Systems, USA	1 : 2000	The rate of positive tumor cells in 10 high power field (400x)/slide: 0–5% low, >5–20% intermediate, and >20% high	20%> versus >20%

D'Alterio, 2010 [17, 18]		240	139/101	61 (26–84)	RCC (chromophobe, papillary, clear cell and other)	Fuhrman grading: I-II/III-IV; 163/58	Tissue sections	Mouse; R&D Systems, USA	1 : 2000	The rate of positive tumor cells in 10 high power field (400x)/slide: 0–5% low, >5–20% intermediate, and >20% high	20%> versus >20%

TS: training set, VS: validation set, M/F: male/female, ccRCC: clear cell RCC, mRCC: metastasis RCC, and LARCC: locally advanced RCC.

**Table 3:** Subgroup analysis based on characteristics of various studies.

Variables	T/P	Overall survival					
		HR (95% CI)	*I*-squared (%)	Model	*P*-He^*∗*^	*Z* value	*P* value
Overall	5/605	2.77 (1.80, 4.27)	51.7	Random effect	0.066	4.61	<0.001
Ethnicity							
Asian	3/439	3.78 (2.47, 5.78)	0.0	Fixed effect	0.60	6.13	<0.001
Non-Asian	2/166	1.68 (1.14, 2.48)	0.0	Fixed effect	0.35	2.63	0.009
Fuhrman grades III-IV (%)							
>70	1/62	1.48 (0.93, 2.37)	NA	NA	NA	1.64	0.102
≤70	4/543	3.26 (2.27, 4.67)	0.0	Fixed effect	0.471	6.41	<0.001
Median follow-up (months)							
>60	2/329	2.70 (1.73, 4.21)	0.0	Fixed effect	0.727	4.39	<0.001
≤60	3/276	3.10 (1.24, 7.80)	78.6	Random effect	0.009	2.41	0.016

T/P: number of trials/number of patients, *P*-He^*∗*^: *P* value of heterogeneity.
